# Levator Ani Necrosis: An Exceptional Complication Occurring after “High Intensity Focused Ultrasound” of the Prostate

**DOI:** 10.1155/2016/3920976

**Published:** 2016-09-08

**Authors:** Danny Badawy, Elie El Rassy, Fouad Aoun, Roland Van Velthoven

**Affiliations:** ^1^Department of Onco-Surgery, Jules Bordet Institute, Brussels, Belgium; ^2^Department of Oncology, Hotel Dieu de France, Saint Joseph University, Beirut, Lebanon; ^3^Department of Urology, Hotel Dieu de France, Saint Joseph University, Beirut, Lebanon; ^4^Department of Urology, Jules Bordet Institute, Brussels, Belgium

## Abstract

High intensity focused ultrasound (HIFU) is a minimally invasive treatment option that might be considered in the management of localized prostate cancer. It is a well-tolerated treatment with few minor urologic complications and no major toxicities. In this paper, we report to our knowledge the first case of levator ani necrosis in a patient treated with HIFU, manifesting as sturdy perineal pain, which took years of NSAID intake and serial MRIs to demonstrate partial improvement. Therefore, we regard HIFU as a serious potential treatment option that still requires longer follow-up data before its approval in the personalized treatment panel of prostate cancer.

## 1. Introduction

In view of the increased incidence of prostate cancer patients, the concept of personalized treatment has been drastically evolving. In the actual state of knowledge, the choice of therapy is based on the tumor stage, prostate-specific antigen (PSA) level, and Gleason score in respect to the patient's age, comorbidities, life expectancy, and preferences [1]. High intensity focused ultrasound (HIFU) is a minimally invasive treatment option that might be considered in the management of localized prostate cancer. This technique was developed in the 1990s to destroy the prostate tissue with high intensity ultrasound waves via a transrectal probe. Pathophysiologically, a coagulation necrosis is induced by the thermal and mechanical effects of the transmitted waves and the prostatic tissue is subsequently destroyed and replaced by a scar tissue within weeks [2]. Overall, HIFU is a well-tolerated treatment without major toxicities. In this paper, we report to our knowledge the first case of levator ani necrosis in a patient treated with HIFU.

## 2. Case Presentation

An 83-year-old asymptomatic male presented to our clinic for an elevated PSA of 8.5 ng/mL. The clinical exam revealed a firm lesion in the left lobe of the prostate. Rectal ultrasound showed a prostate of 25 g. Ultrasound guided transrectal biopsy revealed a Gleason 3 + 4 adenocarcinoma of the prostate. Magnetic Resonance Imaging confirmed the presence of a localized disease. The patient refused to be observed and opted for whole gland HIFU to avoid the collateral damage of more invasive primary treatment such as radical prostatectomy and radiation therapy. Subsequently, the patient was managed with HIFU using Ablatherm technology.

**Figure 1:**
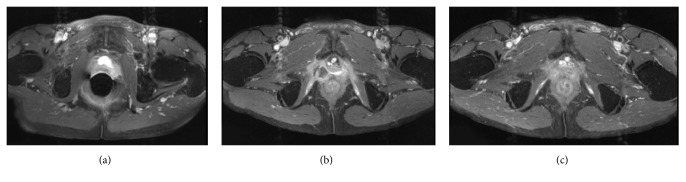
MRI revealing intact levator ani before HIFU (a), marked levator ani necrosis 2 months after HIFU (b), and regression of the levator ani necrosis after 3 years (c).

Two months later, the patient consulted for a perineal pain that increases throughout the day in concordance with alternating episodes of pollakiuria, urinary incontinence, and urinary retention. His physical examination was unremarkable except for a faint intergluteal erythema. A gadolinium-enhanced pelvic MRI showed necrosis of the levator ani muscle bilaterally and more markedly on the right (Figure 1). A bladder ultrasound was normal. The PSA level was 2.17 ng/mL. The patient was managed with symptomatic treatment. One year later, the patient had a marked improvement of the urinary symptoms with only mild improvement of the perineal pain. A nonsteroidal anti-inflammatory drug was also prescribed to help manage the pain. PSA level was 4.13 ng/mL and follow-up PSA levels remained unchanged. The patient was reevaluated at years 3, 4, and 6 after HIFU with PSA at 5.4, 12.0, and 11.0, respectively. Simultaneously, the patient was also followed up with several MRIs (Table 1). The urinary symptoms had almost disappeared by that time, but the perineal pain persisted despite a noted decrease in intensity.

## 3. Discussion

The recommendations concerning HIFU for the treatment of prostate cancer are still conflicting in view of the paucity of data concerning survival and quality of life [3]. The rationale behind its development is the induction of irreversible damage in the localized prostate cancer [4]. The energy deposit can result in boiling the liquids composing the tissue to denaturize the composing proteins and melt the lipid membranes [5]. This would create air pockets that modify the targeted tissue in an uncontrollable way. In cases of excess boiling, the mechanical damage results in bubble formation and cavitation [6]. In the treatment of prostate cancer, HIFU is administered systematically throughout the target volume at different locations [7]. In our case, unfortunately, the energy was deposited inside the levator ani muscle. This is mainly due to the inaccurate monitoring system that is ultrasound used in Ablatherm.

Two HIFU technologies, the Ablatherm and the Sonablate, were available at the moment of treatment of the patient. The differences between the two devices arise in the optimization of frequencies and intensities of the waves [8]. Regarding our case, the patient received HIFU via Ablatherm. This machine includes the patient's bed, ultrasound power generation, probe positioning system, cooling system for preservation of the rectal wall, and ultrasound scanner used during treatment localization phase. The Ablatherm contains a safety ring stabilizing the rectal wall and a motion detector that hampers the waves if the patient moves. The HIFU is administered in a single session of 2 to 3 hours under spinal anesthesia [9]. Despite these safety measures, a review of the literature reported multiple adverse events among patients using HIFU as a primary therapy option in prostate cancer. Adverse events included bladder neck/urethral stenosis/stricture (2–17%), prolonged urinary retention (3–14%), urinary tract infection (2–58%), urinary incontinence (2–34%), rectal burns (0–15%), and rectourethral fistula (0–3%) [10]. In the same setting, our patient had urinary obstructive symptoms in addition to perianal pain caused by a necrosis of the levator ani that eventually resolved while maintaining residual pain. We believe that HIFU is a serious potential treatment option that still requires longer follow-up data before its approval in the personalized treatment panel of prostate cancer. Nowadays, a more sophisticated HIFU technology is available in some oncologic centers. The latter allows better monitoring of the prostate landscape in a real time fashion thanks to its cutting edge technologies. The safety of this device remains to be demonstrated.

## Figures and Tables

**Table 1:** Pelvic MRI follow-up after HIFU.

Time	Prostate	Bladder	Muscles
Before HIFU	Normal size. Normal borders and capsule. Left lobe tumor with abnormal contrast uptake.	Mild parietal hypertrophy	Normal

2 months after HIFU	Small size. Slightly irregular borders. Central necrosis.	Bladder neck enlargement	Levator ani muscle necrosis extending to the right interprostatorectal space

6 months after HIFU	Small size. Slightly irregular borders. Central necrosis.	Normal	Regression of the necrosis between the internal obturator and rectum. Persistence of the necrosis at the level of the left levator ani

3 years after HIFU	Small size. Slightly irregular borders. Central necrosis.	Normal	Normal puborectalis and sphincter muscles. Marked regression of the levator ani necrosis

6 years after HIFU	Abnormal contrast uptake in the left lobe.	Normal	Normal. No sign of levator ani necrosis

HIFU: high intensity focal ultrasound; MRI: magnetic resonance imaging.

## References

[1] Aus G., Abbou C. C., Bolla M. (2005). EAU guidelines on prostate cancer. *European Urology*.

[2] http://www.edap-tms.com/.

[3] Warmuth M., Johansson T., Mad P. (2010). Systematic review of the efficacy and safety of high-intensity focussed ultrasound for the primary and salvage treatment of prostate cancer. *European Urology*.

[4] Linke C. A., Carstensen E. L., Frizzell L. A., Elbadawi A., Fridd C. W. (1973). Localized tissue destruction by high-intensity focused ultrasound. *Archives of Surgery*.

[5] Hill C. R., ter Haar G. R. (1995). High intensity focused ultrasound—potential for cancer treatment. *British Journal of Radiology*.

[6] Curiel L., Chavrier F., Gignoux B., Pichardo S., Chesnais S., Chapelon J. Y. (2004). Experimental evaluation of lesion prediction modelling in the presence of cavitation bubbles: intended for high-intensity focused ultrasound prostate treatment. *Medical and Biological Engineering and Computing*.

[7] Foster R. S., Bihrle R., Sanghvi N. T., Fry F. J., Donohue J. P. (1993). High-intensity focused ultrasound in the treatment of prostatic disease. *European Urology*.

[8] HIFU—what does the treatment involve?. http://sonacaremedical.com/index.php/surgeons/our-products/sonasource.

[9] Chaussy C. G., Thüroff S. (2010). Robot-assisted high-intensity focused ultrasound in focal therapy of prostate cancer. *Journal of Endourology*.

[10] Alkhorayef M., Mahmoud M. Z., Alzimami K. S., Sulieman A., Fagiri M. A. (2015). High-intensity focused ultrasound (HIFU) in localized prostate cancer treatment. *Polish Journal of Radiology*.

